# Betahistine alleviates benign paroxysmal positional vertigo (BPPV) through inducing production of multiple CTRP family members and activating the ERK1/2-AKT/PPARy pathway

**DOI:** 10.1186/s40659-022-00385-3

**Published:** 2022-04-04

**Authors:** Jing Hui, Qi Lei, Zhi Ji, Dingjing Zi

**Affiliations:** 1 Department of Neurology, The Second Affiliated Hospital of Xi’an Medical College, Xi’an, 710038 China; 2 Shaanxi Normal University Hospital, Xi’an, 710119 China; 3 Department of Otolaryngology, The Second Affiliated Hospital of Air Force Medical University, No. 1 Xinsi Road, Xi’an , 710038 China

**Keywords:** BPPV, Betahistine, CTRP family, Vestibular dysfunction, The ERK1/2-AKT/PPARy pathway

## Abstract

**Background:**

Betahistine is a clinical medication for the treatment of benign paroxysmal positional vertigo (BPPV). Otolin, a secreted glycoprotein with a C-terminal globular domain homologous to the immune complement C1q, has been identified as a biomarker for BPPV. However, the role of complement C1q/TNF-related proteins (CTRPs) with a C-terminal globular domain in BPPV is unclear, so we explored the change of CTRPs in betahistine treated BPPV.

**Methods:**

We treated BPPV patients with Betahistine (12 mg/time, 3 times/day) for 4 weeks and observed the clinical efficacy and the expression of CTRP family members in BPPV patients. Then, we constructed a vertigo mice model of vestibular dysfunction with gentamicin (150 mg/Kg) and a BPPV model of *Slc26a4*^*loop/loop*^ mutant mice. Adenoviral vectors for CTRP expression vector and small interfering RNA were injected via the intratympanic injection into mice and detected the expression of CTRP family members, phosphorylation levels of ERK and AKT and the expression of PPARγ. In addition, we treated mice of vestibular dysfunction with Betahistine (10 mg/Kg) and/or ERK inhibitor of SCH772984 (12 mg/Kg) and/or and PPARγ antagonist GW9662 (1 mg/Kg) for 15 days, and evaluated the accuracy of air righting reflex, the time of contact righting reflex and the scores of head tilt and swimming behavior.

**Results:**

After treatment with Betahistine, the residual dizziness duration and the score of the evaluation were reduced, and the expression of CTRP1, 3, 6, 9 and 12 were significantly increased in BPPV patients. We also found that Betahistine improved the accuracy of air righting reflex, reduced the time of contact righting reflex and the scores of head tilt and swimming behavior in gentamicin-treated mice and *Slc26a4*^*loop/loop*^ mutant mice. The expression levels of CTRP1, 3, 6, 9 and 12, phosphorylation levels of ERK and AKT, and PPARγ expression were significantly increased, and the scores of head tilt and swimming behavior were decreased in vestibular dysfunction mice with overexpression of CTRPs. Silencing CTRPs has the opposite effect. SCH772984 reversed the effect of Betahistine in mice with vestibular dysfunction.

**Conclusion:**

Betahistine alleviates BPPV through inducing production of multiple CTRP family members and activating the ERK1/2-AKT/PPARy pathway.

**Supplementary Information:**

The online version contains supplementary material available at 10.1186/s40659-022-00385-3.

## Background

Benign paroxysmal positional vertigo (BPPV) is the most common vestibular system disorder causing peripheral vertigo, accounting for 20% of all vertigo cases [1]. The lifetime prevalence of BPPV was 2.4%, with 10.7—64.0 cases per 100,000 population. It generally emerges in the elderly with a peak onset in the 5th and 6th decade of life [2]. The main pathogenesis of BPPV is detachment of otoconia from the utricular macula migrating into the semicircular canals leading to a change in fluid dynamics of endolymph. While it is mostly seen unilaterally with single canal involvement, more than one semicircular canal may be occasionally affected. Patients present with complaints of sudden episodes of dizziness especially head movements. Despite the disease recovered after spontaneous remission within days or weeks, 50% of patients may relapse [2, 3]. Vestibular disorders have a negative impact on the quality of life of patients, and ultimately lead to the loss of labor capacity [4]. Choosing effective methods to eliminate the disease is very important to improve the quality of life of BPPV patients.

In recent years, researchers have been focusing on the development of drugs or treatment methods for the treatment of BPPV patients, such as dexamethasone [5], antianxiety drugs (low-dose of etizolam) [6], Danhong injection [7] and Epley manoeuvre [8]. Betahistine has been developed as a marketed drug and is commonly used to treat vertigo symptoms caused by different reasons [9]. For example, CASANI et al.’s study showed that Betahistine was used in the intercritical phase of Ménière’s disease to reduce the number and severity of vertigo attacks [10]. Chen et al. reported that combined Betahistine and puerarin regimens were more effective in treating vertebrobasilar artery ischemic vertigo compared with other, conventional drugs; effectively alleviating the associated symptoms, including dizziness and increased average blood flow velocity within the vertebrobasilar arteries, without causing an increased number of serious side effects [11]. Li et al.’s study showed that Oral Betahistine mesylate 12 mg twice daily improved the patient's cervical vertigo [12]. There are also reports that Betahistine can alleviate BPPV, for example Betahistine (6 mg/time, three times/day) plus cognitive behavioral therapy could reduce vertigo in BPPV patients [13], Betahistine and Epley manoeuvre could effectively reduce vertigo and improve the quality of life of BPPV patients [8].

C1q tumor necrosis factor (TNF) related proteins (CTRPs) has been identified as a highly conserved family of adiponectin paralogs. CTRP proteins share a common structure composed of four distinct domains, including a signal peptide at the N terminus, a short variable domain, a collagen-like domain, and a C-terminal globular domain that is homologous to complement component 1q (C1q). It has been reported that Otolin, a secreted glycoprotein, has a C-terminal globular domain homologous to the immune complement C1q, that could regulate BPPV and is a biomarker for BPPV. However, it is still not determined whether the CTRP family play the roles in BPPV. In addition, members of the CTRP family play a biological role by activating multiple signaling pathways, such as Chi1 et al. [14] founds adipokine CTRP6 could alleviate hypertension and vascular endothelial dysfunction in spontaneously hypertensive mice through activating PPARγ. Hironori et al. [15] showed that CTRP1 regulates chondrocyte proliferation and maturation through the ERK1/2 pathway. Xiang et al. [16] showed that CTRP6 attenuates renal ischemia–reperfusion injury through activating the PI3K/AKT pathway. Hence, the purpose of this study is to investigate whether CTRPs play a role in the treatment of BPPV with Betahistine, and the underlying mechanism.

## Materials and methods

### BPPV diagnosis and treatment

After a thorough physical examination and a review of clinical history, each candidate underwent the supine head-turning test or Dix-Hallpike test. In most cases (about 90%), BPPV was involved with the posterior semicircular canal, and most patients were diagnosed with canalithiasis type. Thus, only the canalithiasis type of BPPV patients involved with the posterior semicircular canal in this study. Sixty patients who were included were randomly assigned to two groups. One group was treated with Betahistine (12 mg/time, 3 times/day); the other group was treated with placebo. This treatment was continued for 4 weeks. Blood samples were then drawn at the end of 4 weeks. All patients signed an informed consent form approved by the second affiliated hospital of air force medical university Institutional Review Board prior to the first blood sampling.

### Measurement index

The primary outcomes were the duration of residual dizziness and scores of the 25-item Dizziness Handicap Inventory (DHI) [17, 18] and the Berg balance scale (BBS) [19]. All patients answered the questionnaires after 4 weeks of treatment. The duration of residual dizziness was used to assess the efficacy of Betahistine treatment on improving the residual dizziness.

The DHI score was used to help patients to rate the dizziness-related physical impairments, activity limitations, and restrictions in participation [17, 18]. The DHI divided into three subdomains of impact on daily life: functional, emotional and physical (Additional file 1: Table S1). Patients were asked to complete the Dizziness Handicap Inventory (DHI) consisting of 25 questions, and a total score (0–100 points) is obtained by summing ordinal scale responses, higher scores indicating more severe handicap. The 25 items were grouped into three dimensions: functional, emotional, and physical aspects of dizziness and unsteadiness. There were nine questions within each of the functional and emotional dimensions; with a maximum score of 4 for each item. Within the physical dimension there were seven questions with a maximum score of 4 for each.

BBS is a valid tool to estimate balance condition. BBS comprises 14 balance-related tasks [19, 20]. The scale requires 15 min to complete and measures the patient's ability to maintain balance for a specified duration, either statically or while performing various functional movements. The items are scored from 0 to 4, with a score of 0 representing an inability to complete the task and a score of 4 representing independent item completion. A global score is calculated out of 56 possible points. Scores of 0 to 20 represent balance impairment, 21 to 40 represent acceptable balance, and 41 to 56 represent good balance. The BBS measures both static and dynamic aspects of balance.

### Establishment and treatment of vestibular function vertigo model

Four-week-old Kunming SPF mice (25 ± 0.5 g), *Slc26a4*^*loop/loop*^ mutant and wild-type mice (25 ± 0.5 g) were purchased from the Experimental Animal Center of the Air Force Military Medical University (Xi’an, China). The mice were kept under controlled conditions at a temperature of 22 ℃ and humidity of 70%, with a 12-h light–dark cycle, and allowed free access to food and water. All experimental animal protocols were reviewed and approved by the Ethics Committee of the second affiliated hospital of air force medical university for the use of laboratory animals.

A total of 70 mice were randomly divided into five groups, including the control group, gentamicin group, Betahistine group and Betahistine + SCH772984 group. Mice of gentamicin group were subcutaneously injected with gentamicin (150 mg/Kg); mice of Betahistine group were subcutaneously injected with gentamicin (150 mg/Kg) and Betahistine (10 mg/Kg); mice of Betahistine + SCH772984 group were subcutaneously injected with gentamicin (150 mg/Kg), Betahistine (10 mg/Kg) and SCH772984 (12 mg/Kg); mice of Betahistine + GW9662 group were subcutaneously injected with gentamicin (150 mg/Kg), Betahistine (10 mg/Kg) and GW9662 (1 mg/Kg). Mice of the control group were subcutaneously injected with normal saline according to 0.10 mL/Kg body weight. The drug was diluted with normal saline and injected for 15 days.

### Establishment and treatment of BPPV model mice

A report showed that pathological findings of displaced otoliths were consistently found in the inner ear of *Slc26a4*^*loop/loop*^ mutant mice with severe vestibular dysfunction, a mouse model with a genetic predisposition for ectopic otolithiasis with clinical relevance to BPPV, and this unique mouse model may serve as a platform for further studies of BPPV pathophysiology and for the development of novel therapeutic approaches in live animal models [21]. So, in our study, we constructed severe vestibular dysfunction *Slc26a4*^*loop/loop*^ mutant mice as a BPPV model.

Thirty-six *Slc26a4*^*loop/loop*^ mutant mice were randomly divided into two groups, including *Slc26a4*^*loop/loop*^ group and Betahistine group. Mice of Betahistine group were subcutaneously injected with Betahistine (10 mg/Kg). *Slc26a4*^*loop/loop*^ mutant mice and wild-type mice were subcutaneously injected with normal saline according to 0.10 mL/Kg body weight. The drug was diluted with normal saline and injected for 15 days.

### Preparation and delivery of adenoviral vectors in vivo

Adenoviral vectors containing the enhanced green fluorescent protein gene were purchased from Life Technologies (Shanghai, China). CTRP-overexpression recombinant adenoviruses (up-Ad-CTRP, 2.0 × 10^10^ pfu/mL) and blank control overexpression recombinant adenoviruses (up-Ad-GFP, 1.1 × 10^11^ pfu/mL), interfering recombinant adenoviruses (down-Ad- CTRP, 1.1 × 10^11^ pfu/mL), and blank control interfering recombinant adenoviruses (down-Ad-GFP, 5 × 10^10^ pfu/mL) were provided by Sangon Biotech (Shanghai) Co., Ltd. (Shanghai, China). Then, 20 μL of adenovirus solution (1 × 10^7^ particles/μL) were injected via the intratympanic injection into designated group.

### Auditory brainstem response (ABR) testing

Auditory brainstem response (ABR) was evaluated as previously described [22]. We tested only young mice (8–14 weeks old) with *Slc26a4*^*loop/loop*^ mutant mice and wild-type mice to preclude complications from the possible age-related hearing loss. A broad-band click stimulus or pure tone pips, at 8, 16 and 32 kHz each, produced by computer (Tucker Davies) was delivered using headphones (Intelligent Hearing Systems) with an attached plastic tube to channel the sound into the ear of the mouse, which had been anaesthetized with avertin (tribromoethylic alcohol/tert-amylic alcohol) at 3.5 mg/10 g of body weight. Mice were kept warm on a heating pad during ABR recording, and tested in a chamber designed for compartmental assessment (Ugo Basile). Subdermal electrodes were inserted behind the ear pinna of the side to be recorded (active), on the top of the head (reference) and at the front of the head (ground). The unrecorded ear was plugged with modelling clay. We analyzed both ears. The ABR threshold is the lowest intensity producing a recognizable ABR pattern on the computer screen (that is, at least two peaks) using 250 stimuli. We determined thresholds for each ear. The maximum intensity of stimulation was 100 dB SPL.

### Behavioral testing

To evaluate vestibular function of *Slc26a4*^*loop/loop*^ mutant mice and wild-type mice, we utilized a battery of behavioral tests with standardized scoring systems as previously described [21]. For the swimming test, the mouse was gently inserted into a bath of warm water and watched carefully. Normal swimming performance were scored with zero, demonstrating regular performance. Mice with irregular swimming were scored with one point, while mice with immobile floating were scored with two points. Mice with an inability to swim were rescued immediately to avoid drowning and ranked with the maximum score of three points. For the trunk curl test, the mouse was gently hung from his tail 10 cm above a clean surface with a metal grid. Normal behavior was demonstrated by a reaching position, with a score of zero, by the extension of limb and head forward and downward aiming to the floor. Mice with an abnormal behavior, ranked with a score of 1, tried to climb towards the examiner s hand, curling the body upward reaching with its head to the tail. The remaining three behavioral tests were based on observation of each mouse in a clean cage. A positive circling behavior was scored with 1 point, while a head tilt gained another point. For gait observation, we defined a scale of zero (normal gait) to three points (incapacity) for mice unable to walk with a typical habitus of a tilted trunk from head to tail lying on the cage floor. All mice in this study were tested between 8 10 weeks of age. The score of each test was normalized and a total sum of the cumulative score was calculated upon completion of the behavioral pipeline, giving each test 20 percent of the final score.

### Evaluation of vestibular function

The vestibular system is known as the balance organs of the inner ear, constituting three roughly orthogonal semicircular canals that sense rotational movements, as well as two otolith organs (the utricle and the saccule) that sense gravity and linear accelerations [23]. As part of the vestibular system, the otoconial membrane and the internal organic matrix of otoconia consist of glycoproteins and sulfated glycosaminoglycans complexes in the form of proteoglycans [24]. Otoconia are crucial for correct processing of orientation and positional information. Mice lacking otoconia cannot sense the direction of the gravity vector and cannot swim properly [25]. In our study, assessment of vestibular function was performed with three tests as previously reported, including the air righting reflex test, the time of contact righting reflex test and the swimming test [25–27].

The air righting reflex test is an antigravity reflex test in mice that depend on vestibular function [27]. Mice were supine and dropped from a height of 50 cm onto a soft filling surface. The average percentage of trials each mouse landed on all four feet was determined from five attempts for each mouse.

The time of contact righting reflex test was performed, and the mice were placed in a 60 mL opaque plastic syringe so that they could roll over but could not turn around or rear. The syringe was inverted until the animal was in the supine position, and the average time for each mouse to recover the upright position within the syringe in three trials was determined.

In the swimming test, the mice were placed in a 20 × 40 cm plexiglass cage filled with water at room temperature for 60 s. In three trials in each mouse, the average swimming time with the head above water was measured.

### Quantitative real-time polymerase chain reaction (RT-qPCR)

Total RNA isolation was performed using the Trizol reagent (Invitrogen, Carlsbad, CA, U.S.A.) according to the manufacturer’s instructions. According to gene sequences published in GenBank’s database, primers were designed using Primer 5.0 design software and synthesized by Shanghai GenePharma Co., Ltd. (Shanghai, China). Reverse transcription was performed with approximately 0.5 μg of total RNA into cDNA with the Superscript™ reverse transcription system (Takara, Dalian, Japan). Aliquots of cDNA were used for PCR amplification with primer-specific. RT-qPCR reaction was performed as follows: 95 ℃ for 3 min, 35 cycles of denaturation at 95 ℃ for 30 s, 58 ℃ for 45 s, and 72 ℃ for 30 s and 8 min at 72 ℃ using SYBR Green PCR master mix reagents (Takara, Dalian, Japan) in the ABI StepOne Plus Real-time PCR system. The relative quantification of mRNA expression levels was calculated using the 2^ − ΔΔCT^ method.

### Western blotting

Total protein was extracted from the inner ear tissues by using ice-cold RIPA buffer (Sigma, St. Louis, MO, USA). Proteins were separated by 12% sodium dodecyl sulfate–polyacrylamide gel electrophoresis (SDS-PAGE) gels and transferred to PVDF membranes (Sigma, St. Louis, MO, USA). The PVDF membranes were blocked with 5% skim milk at room temperature for 2 h. Then, the membranes were placed into the primary antibody solution for incubation at 4 ℃ overnight. The membranes were washed 3 times/5 min with TBST and placed into the secondary antibody solution for incubation at room temperature for 1 h and rewashed 3 times/5 min with TBST. The band development of protein was visualized by using the ECL chemiluminescence reagent at a chemiluminescent detection system, and the grey values of target proteins were performed image analysis by using Image J software. Antibodies used in this study are as follows: CTRP1 (1:600), CTRP3 (1:600), CTRP6 (1:600), CTRP9 (1:600), CTRP12 (1:600), ERK1/2 (1:1000), AKT (1:1000), PPARγ (1:1000) and β-actin (1:1000). All primary antibodies were purchased from Abcam, Cambridge, MA, USA.

### ELISA assay

Blood samples were centrifuged at 4 ℃ for 15 min at 3600 rpm to separate serum and analyzed CTRP1, 2, 3, 4, 5, 6, 7, 9, 10, 11, 12, 13, 15 and adiponectin levels in serum with the ELISA kit, according to the manufacturer’s instructions. All ELISA kits were purchased from AdipoGen Life Sciences.

### Statistical analysis

All data are shown as the mean ± SEM of three or more independent experiments were performed. Statistical differences were evaluated by using a one-way analysis of variance (ANOVA) and the Student’s t-test. In the univariate analysis, comparisons of numerical parameters between both groups were evaluated by using the Student’s t-test, and multigroup comparisons of the means were evaluated by using a one-way ANOVA test. Values were considered statistically significant differences at *P* < 0.05. Statistics were performed with SPSS Statistics version 20.0 software.

## Results

### Clinical effect of Betahistine on BPPV

**Fig. 1:**
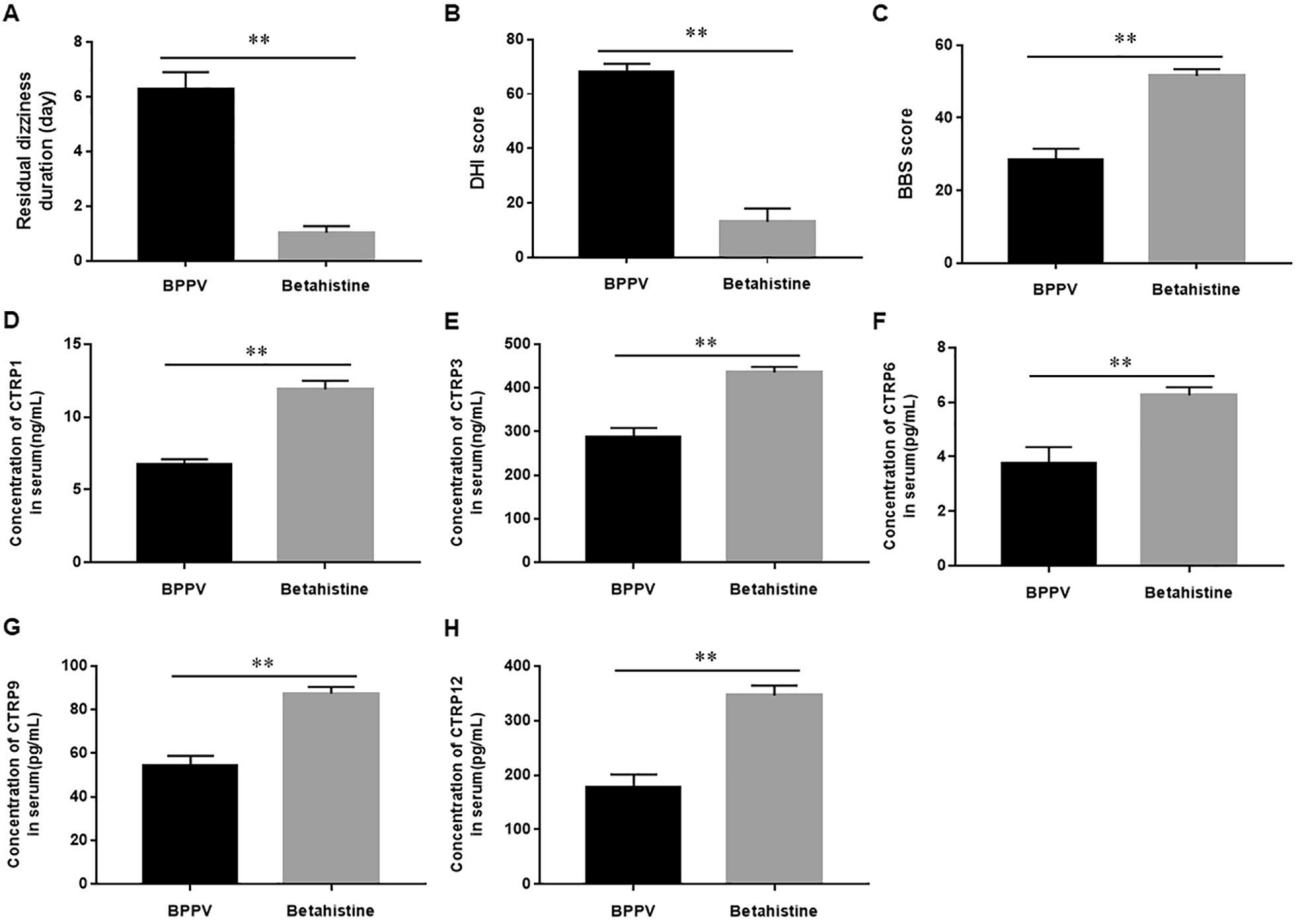
The clinical effect of Betahistine on BPPV and the expression of multiple CTRP family members. BPPV patients were treated with Betahistine (12 mg/time, 3 times/day) for 4 weeks, and blood was collected and serum was separated. The duration of residual dizziness (**A**), DHI score (**B**) and BBS score (**C**) were recorded. The expression of CTRP1 (**D**), CTRP 3 (**E**), CTRP 6 (**F**), CTRP 9 (**G**) and CTRP 12 (**H**) in serum was detected by using ELISA. N = 5, tatistical differences were performed by using the Student’s t-test. Compared with BPPV patient, *P < 0.05, **P < 0.01

To evaluate the efficacy of Betahistine in patients with BPPV, we treated BPPV patients with Betahistine for 4 weeks. We observed that the duration of dizziness was significantly shortened in Betahistine-treated BPPV patients compared to placebo treated BPPV patients (Fig. 1A). In addition, we also found significant decreases in the score of DHI (Fig. 1B) and BBS (Fig. 1C) in BPPV patients treated with Betahistine. These results suggest that Betahistine effectively alleviates the symptoms of BPPV patients in clinical practice.

### Betahistine induces the expression of multiple CTRP family members in BPPV

To explore the regulatory mechanism of Betahistine on BPPV, we examined the expression of CTRP family members in BPPV. We observed that serum CTRP 1, 2, 3, 4, 5, 6, 7, 9, 10, 11, 12, 13, 15 and adiponectin levels in BPPV patients were increased after Betahistine treatment, while the changes of CTRP1, 3, 6, 9 and 12 levels had greater significance after Betahistine treatment (Fig. 1D–H and Additional file 2: Figure S1 A–I). Therefore, we speculate that the protective effect of Betahistine in BPPV may be achieved by inducing the expression of CTRP family members. We selected markedly changed CTRP1, 3, 6, 9 and 12 for the next studies.

### Construction of a vertigo model of vestibular dysfunction in mice

**Fig. 2:**
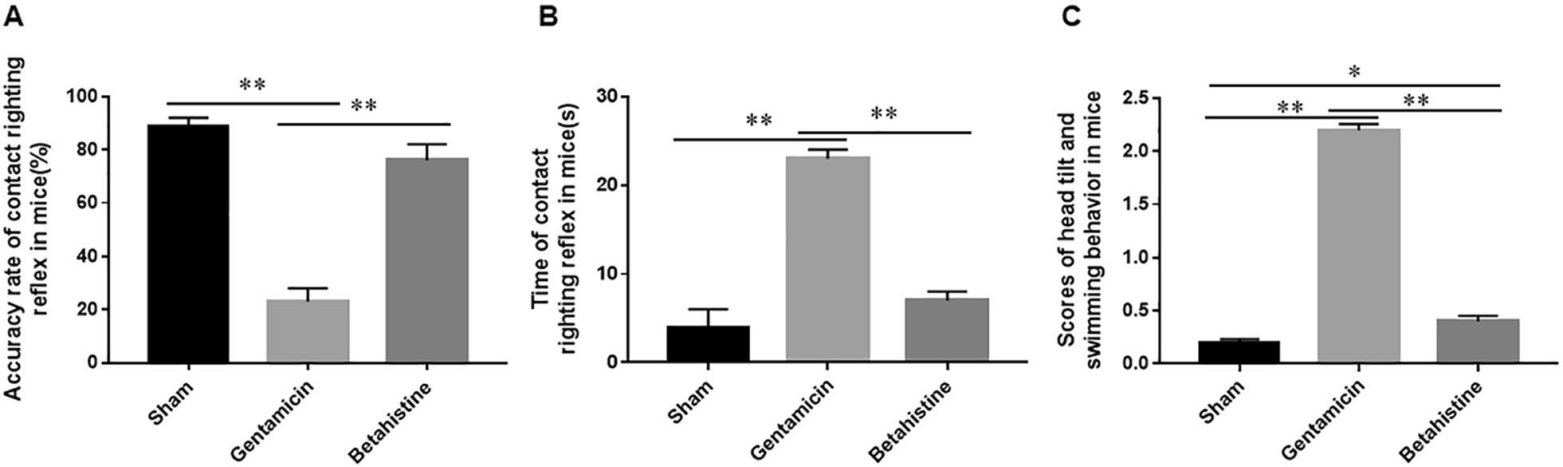
Construction of a vertigo model of vestibular dysfunction in mice. Mice were subcutaneously injected with gentamicin (150 mg/Kg) and/or Betahistine (10 mg/Kg) for 15 days, twice a day. We evaluated the accuracy of air righting reflex (**A**), the time of contact righting reflex (**B**) and the scores of head tilt and swimming behavior (**C**) in vertigo mice of vestibular dysfunction. N = 7, Statistical differences were performed by using a one-way ANOVA. *P < 0.05, **P < 0.01

To further explore the mechanism of Betahistine on BPPV, we constructed a vertigo model of vestibular dysfunction by gentamicin injection in mice. We recorded the accuracy of air righting reflex, the time of contact righting reflex and the scores of head tilt and swimming behavior in mice. We found that the accuracy of air righting reflex was decreased (Fig. 2A), the time of contact righting reflex was prolonged (Fig. 2B) and the scores of head tilt and swimming behavior were significantly increased in mice treated with gentamicin (Fig. 2C). Betahistine counteracted the effects of gentamicin on mice and improved vestibular dysfunction in mice.

### Overexpression of CTRP1, 3, 6, 9 and 12 reduces vestibular dysfunction in mice

**Fig. 3:**
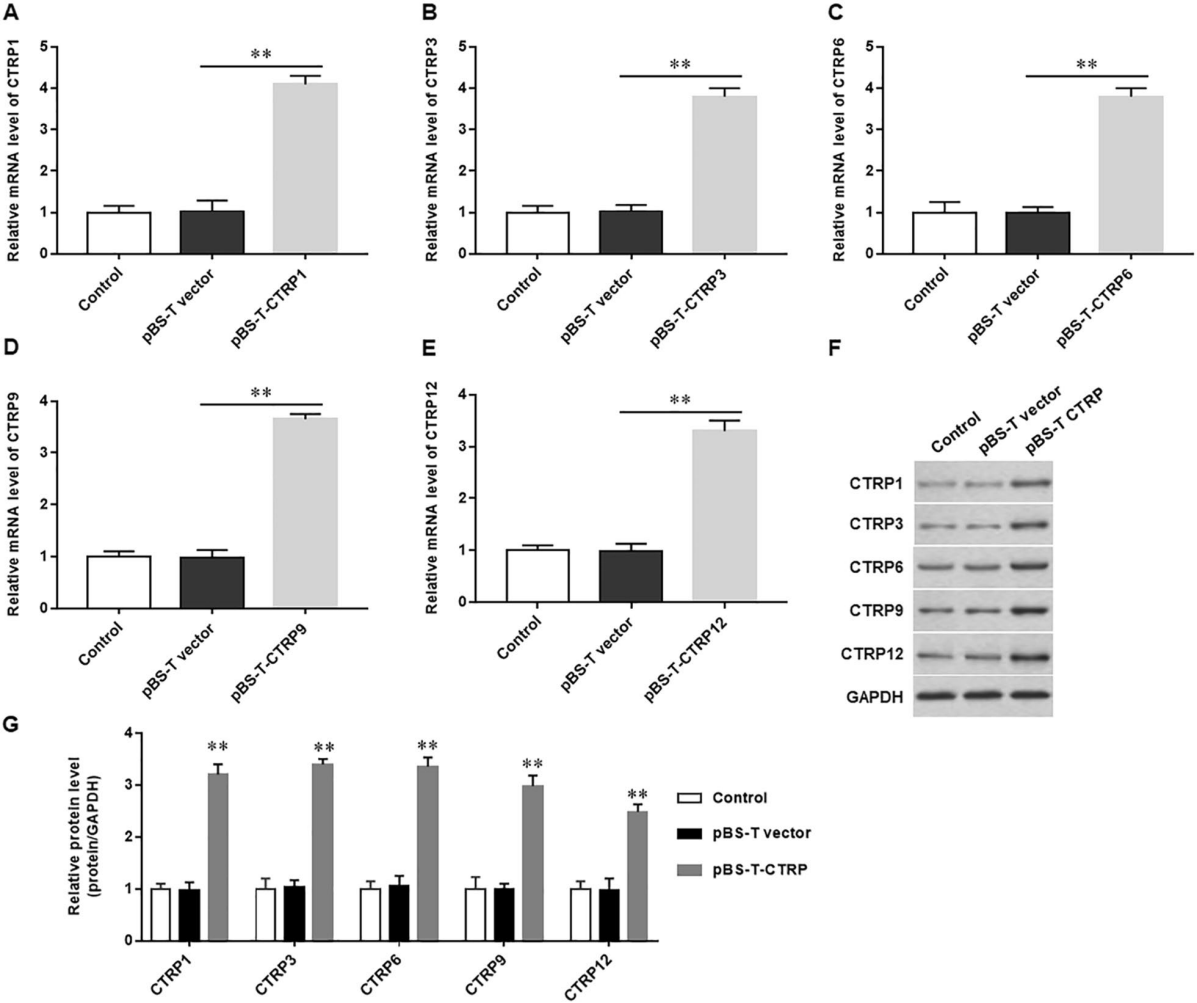
Overexpression of CTRP1, 3, 6, 9 and 12 in mice with vestibular dysfunction vertigo. Mice of vestibular dysfunction vertigo were injected with pSB-T-CTRP1, 3, 6, 9, 12 or pSB-T empty vector, respectively. RT-qPCR was used to detect the mRNA expression of CTRP1 (**A**), CTRP3 (**B**), CTRP6 (**C**), CTRP9 (**D**) and CTRP12 (**E**). **F** and **G** Western blotting was used to evaluate the protein expression levels of CTRP1, 3, 6, 9 and 12. N = 4, Statistical differences were performed by using the Student’s t-test. *P < 0.05, **P < 0.01

**Fig. 4:**
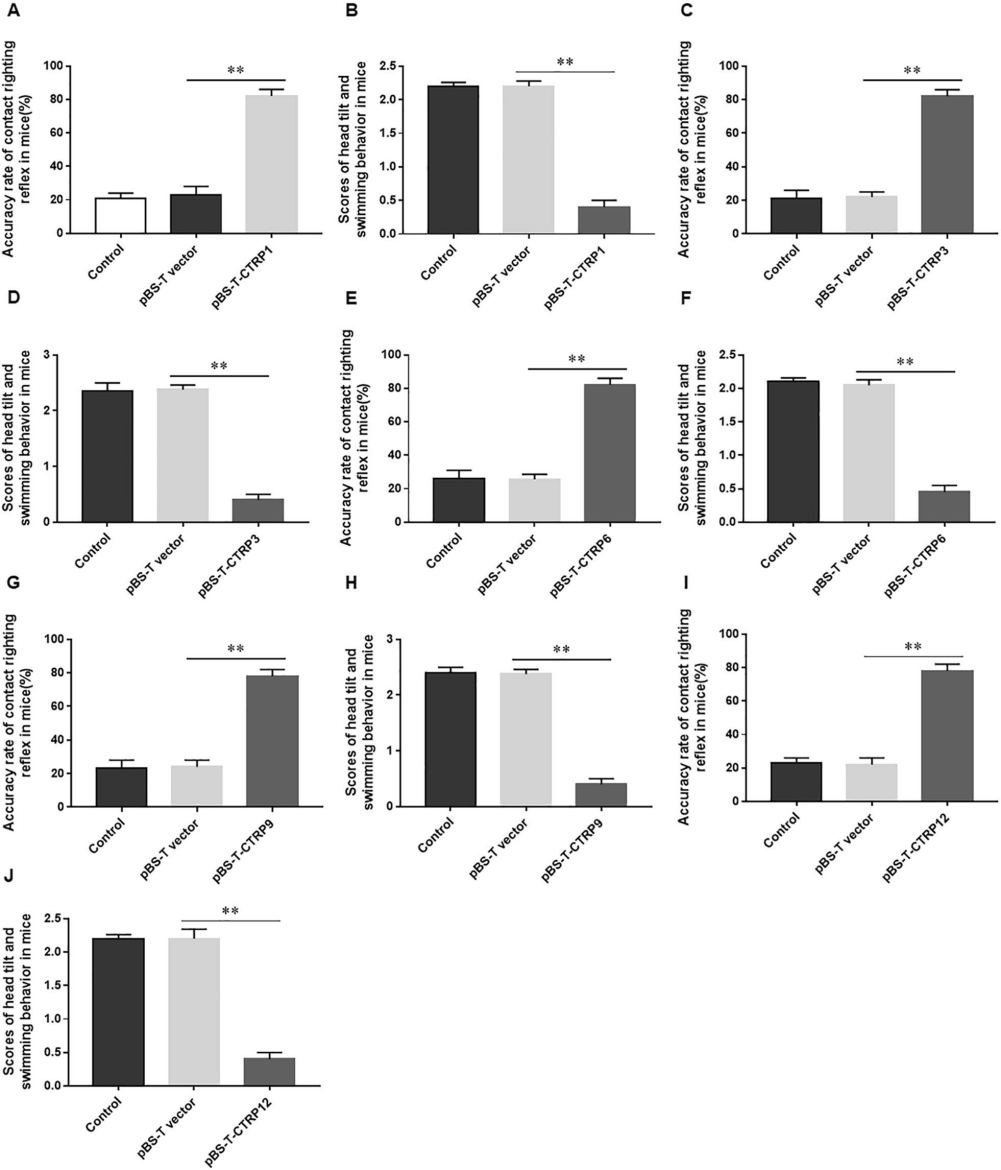
Overexpression of CTRP1, 3, 6, 9 and 12 reduces vestibular dysfunction in mice. The accuracy of air righting reflex and the scores of head tilt and swimming behavior were detected in mice treated with pSB-T-CTRP1 (**A** and **B**), pSB-T-CTRP3 (**C** and **D**), pSB-T-CTRP6 (**E** and **F**), pSB-T-CTRP9 (**G** and **H**) and pSB-T-CTRP12 (**I** and **J**), respectively. N = 3, Statistical differences were performed by using the Student’s t-test. *P < 0.05, **P < 0.01

After subcutaneous injection of pBS-T-CTRP1, 3, 6, 9 and 12, respectively, we observed that the expression of CTRP1 (Fig. 3A), CTRP3 (Fig. 3B), CTRP6 (Fig. 3C), CTRP9 (Fig. 3D) and CTRP12 (Fig. 3E) mRNA levels and protein levels (Fig. 3F and G) were significantly increased. In addition, we also found that the accuracy of air righting reflex was increased, and the scores of head tilt and swimming behavior were significantly decreased in mice treated with pSB-T-CTRP1 (Fig. 4A and B), pSB-T-CTRP3 (Fig. 4C and D), pSB-T-CTRP6 (Fig. 4E and F), pSB-T-CTRP9 (Fig. 4G and H) and pSB-T-CTRP12 (Fig. 4I and J), respectively. These results indicate that overexpression of CTRP family members alleviates vestibular dysfunction in mice.

### Silencing CTRP1, 3, 6, 9 and 12 exacerbates vestibular dysfunction in mice

**Fig. 5:**
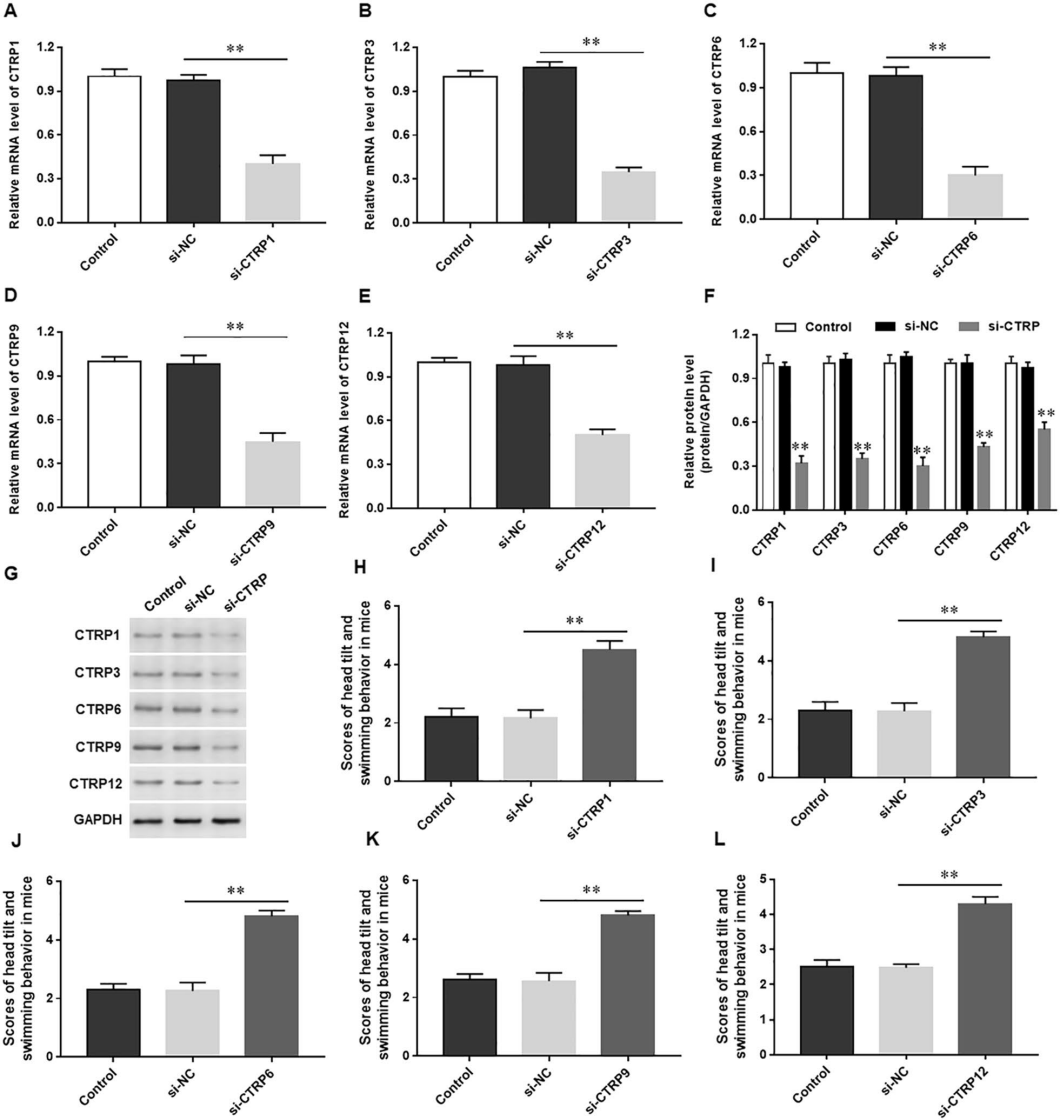
Silencing CTRP1, 3, 6, 9 and 12 exacerbates vestibular dysfunction in mice. Mice of vestibular dysfunction vertigo were injected with si-CTRP1, 3, 6, 9, 12 or their scrambled, respectively. RT-qPCR was used to detect the mRNA expression of CTRP1 (**A**), CTRP3 (**B**), CTRP6 (**C**), CTRP9 (**D**) and CTRP12 (**E**). **F** and **G** Western blotting was used to evaluate the protein expression levels of CTRP1, 3, 6, 9 and 12. The scores of head tilt and swimming behavior were detected in mice treated with si-CTRP1 (**H**), si-CTRP3 (**I**), si-CTRP6 (**J**), si-CTRP9 (**K**) and si-CTRP12 (**L**), respectively. N = 3, Statistical differences were performed by using the Student’s t-test. *P < 0.05, **P < 0.01

To further verify the regulatory effect of CTRP family members in mice with vestibular dysfunction vertigo, we injected siRNA into mice to silence CTRP family members. We found that the expression of CTRP1 (Fig. 5A), CTRP3 (Fig. 5B), CTRP6 (Fig. 5C), CTRP9 (Fig. 5D) and CTRP12 (Fig. 5E) mRNA levels and protein levels (Fig. 5F and G) were significantly decreased, the scores of head tilt and swimming behavior were significantly increased in mice treated with si-CTRP1 (Fig. 5H), si-CTRP3 (Fig. 5I), si-CTRP6 (Fig. 5J), si-CTRP9 (Fig. 5K) and si-CTRP12 (Fig. 5L), respectively. These results confirmed the negative regulatory effect of CTRP family members on vestibular dysfunction in mice.

### Overexpression of CTRP1 and 12 promotes phosphorylation of ERK1/2 and AKT and the expression of PPARγ

**Fig. 6:**
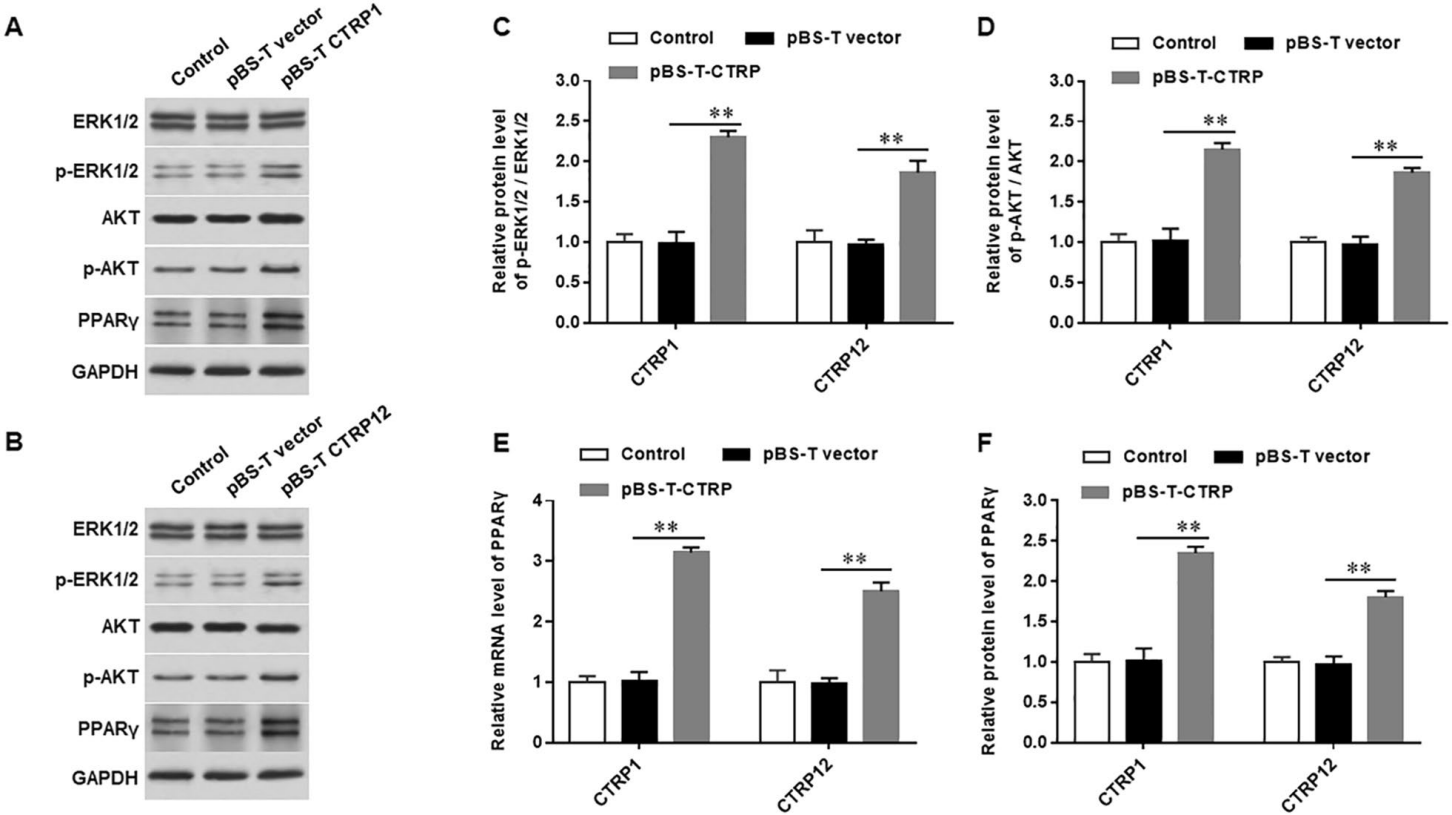
Overexpression of CTRP1 and 12 promotes phosphorylation of ERK1/2 and AKT and expression of PPARγ. Mice of vestibular dysfunction vertigo were injected with pSB-T-CTRP1, 12 or pSB-T empty vector, respectively. **A** and **B** We detected the protein levels of p-ERK1/2 (**C**) and p-AKT (**D**) by using western blotting and evaluated the mRNA (**E**) and protein expression levels of PPARγ (**F**). N = 3, Statistical differences were performed by using the Student’s t-test. *P < 0.05, **P < 0.01

The phosphorylation level of ERK1/2 and AKT was increased significantly (Fig. 6A–D), the mRNA (Fig. 6E) and protein expression level (Fig. 6F) of PPARγ was upregulated in mice of vestibular dysfunction vertigo treated with pBS-T-CTRP1 and pBS-T-CTRP12, respectively. Based on these results, we speculated that overexpression of CTRP family members might activate the ERK1/2-AKT/PPARγ signaling pathway. Previous reports showed that of the C1q family members, CTRP1-15 share strikingly similar structural organization and biochemical properties with adiponectin, and that they exert biological activity through this structural organization [28, 29]. Based on these results, we speculated that CTRP family members have similar effects and their overexpression may be consistent with the results of overexpressing CTRP1 and 12 that could activate the ERK1/2-AKT/PPARγ signaling pathway.

### Betahistine reduces vestibular dysfunction in mice through inducing the expression of CTRP family members and activating the ERK1/2-AKT/PPARγ pathway

**Fig. 7:**
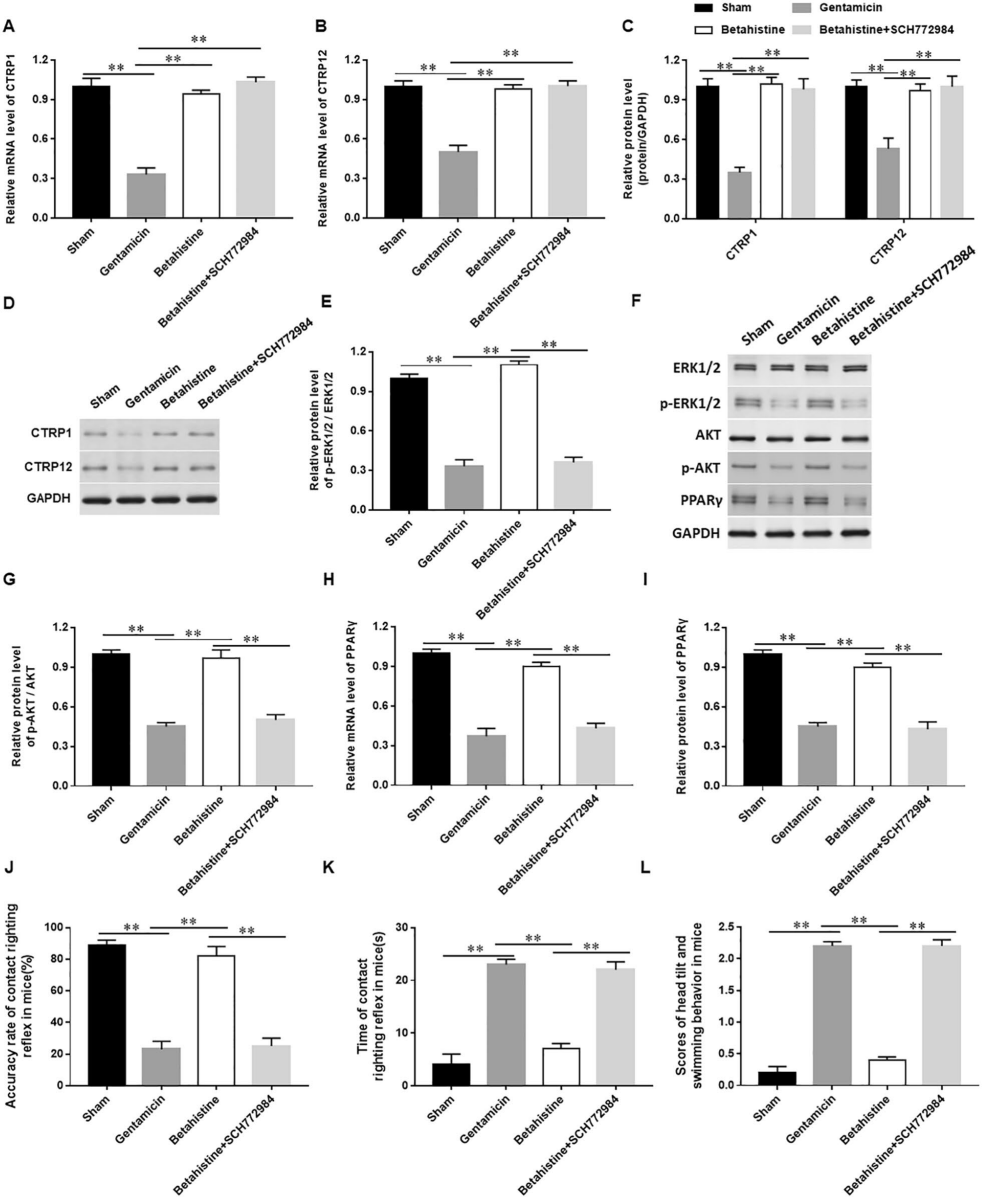
Betahistine reduces vestibular dysfunction through inducing the expression of CTRP family members and activating the ERK1/2-AKT/PPARγ pathway in mice. Fifty-six mice were randomly divided into four groups and treated with normal saline, gentamicin, gentamicin + Betahistine, gentamicin + Betahistine + SCH772984, respectively. Then, CTRP1 (**A**) and CTRP12 mRNA (**B**) and protein levels (**C** and **D**), p-ERK1/2 protein levels (**E** and **F**), p-AKT protein levels (**G**) and PPARγ mRNA (**H**) and protein levels (**I**) were detected with RT-qPCR and western blotting, respectively. We also evaluated the accuracy of air righting reflex (**J**), the time of contact righting reflex (**K**) and the scores of head tilt and swimming behavior in mice (**L**). N = 7, Statistical differences were performed by using a one-way ANOVA. *P < 0.05, **P < 0.01

A total of 70 mice were randomly divided into five groups and treated with normal saline, gentamicin, gentamicin + Betahistine, gentamicin + Betahistine + SCH772984, gentamicin + Betahistine + GW9662, respectively. Compared with mice in the control group, the mRNA (Fig. 7A and B) and protein expression level (Fig. 7C and D) of CTRP1 and 12 was significantly decreased, and the phosphorylation level of ERK1/2 (Fig. 7E and F) and AKT (Fig. 7G) and PPARγ mRNA (Fig. 7H) and protein expression (Fig. 7I) was reduced in mice treated with gentamicin. Betahistine significantly increased the accuracy of air righting reflex (Fig. 7J), shortened the time of contact righting reflex (Fig. 7K) and decreased the scores of head tilt and swimming behavior (Fig. 7L) in mice treated with gentamicin. Furthermore, we observed that ERK1/2 inhibitor SCH772984 and PPARγ antagonist GW9662 counteracted the effect of Betahistine, but had no effect on the expression of CTRP1 and CTRP12 in mice with vestibular dysfunction vertigo (Fig. 7A–L and Additional file 2: Figure S2 A–E). From this, we determined that Betahistine reduces vestibular dysfunction in mice through inducing the expression of CTRP family members and activating the ERK1/2-AKT/PPARγ pathway.

### The changes of CTRPs in Betahistine treated *Slc26a4*^*loop/loop*^ mutant mice

**Fig. 8:**
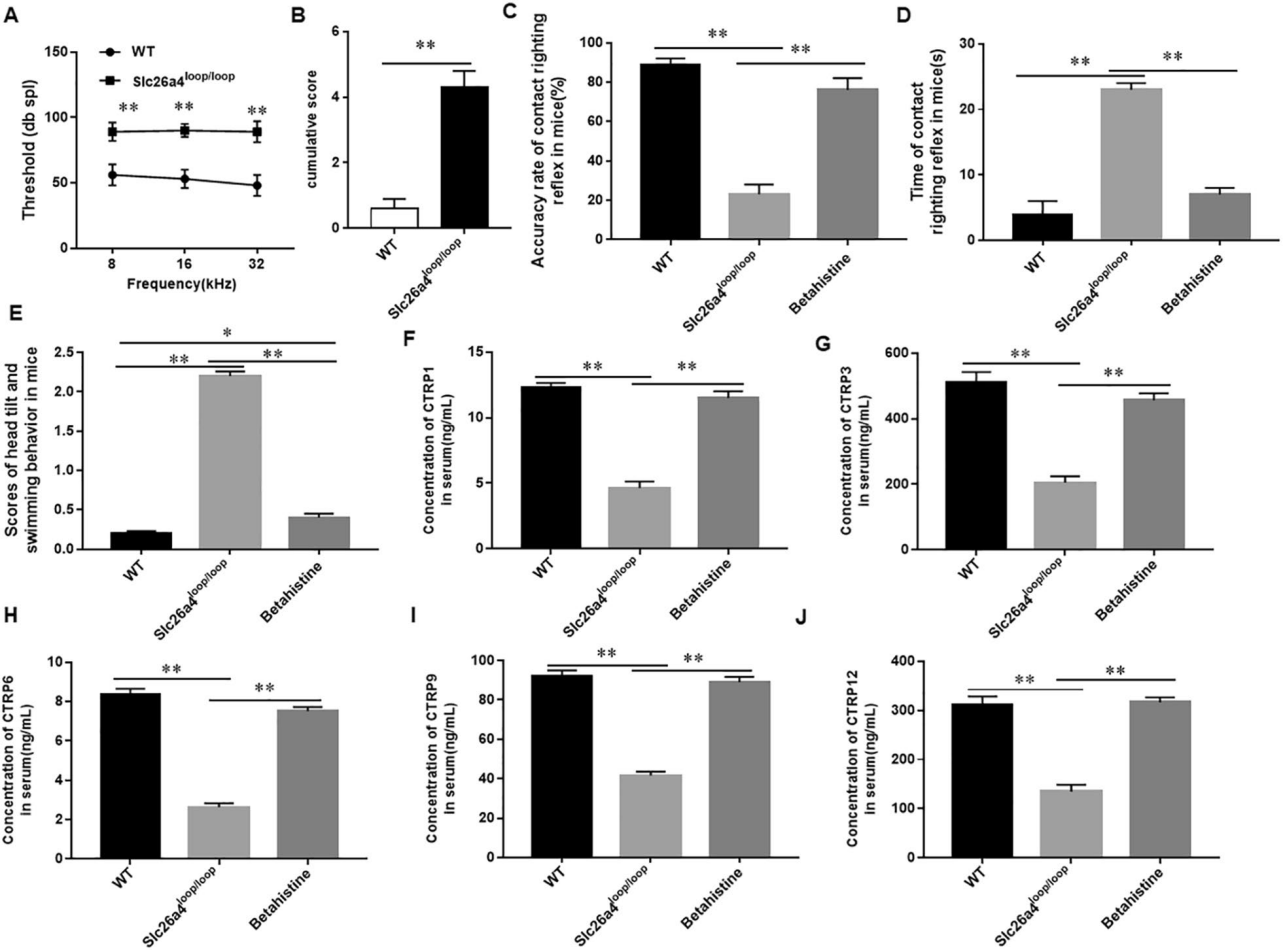
The expression of CTRPs in Betahistine treated *Slc26a4*^*loop/loop*^ mutant mice. *Slc26a4loop/loop* mutant mice were subcutaneously injected with Betahistine (10 mg/Kg) for 15 days, twice a day. We performed auditory brainstem response (ABR) testing (**A**) and behavioral testing (**B**) in *Slc26a4*^*loop/loop*^ mutant and wild-type mice. Next, we evaluated the accuracy of air righting reflex (**C**), the time of contact righting reflex (**D**) and the scores of head tilt and swimming behavior in vertigo mice of vestibular dysfunction (**E**). The expression of CTRP1 (**F**), CTRP 3 (**G**), CTRP 6 (**H**), CTRP 9 (**I**) and CTRP 12 (**J**). In serum was detected by using ELISA. N = 7, Statistical differences were performed by using one-way ANOVA. *P < 0.05, **P < 0.01

We found by auditory brainstem response (ABR) testing and behavioral testing that all *Slc26a4*^*loop/loop*^ mutant mice were profoundly deaf and had severe vestibular dysfunction (Fig. 8A and B). Those indicated that we successfully obtained the BPPV mouse model. Next, we recorded the accuracy of air righting reflex, the time of contact righting reflex and the scores of head tilt and swimming behavior in mice. We found that the accuracy of air righting reflex was decreased, the time of contact righting reflex was prolonged and the scores of head tilt and swimming behavior were significantly increased in *Slc26a4*^*loop/loop*^ mutant mice (Fig. 8C–E). Betahistine improved the behavioral disorders in *Slc26a4*^*loop/loop*^ mutant mice. In addition, we detected the CTRPs expression in Betahistine treated *Slc26a4*^*loop/loop*^ mutant mice. The results showed that the expression of CTRP1, 3, 6, 9 and 12 was significantly downregulated in *Slc26a4*^*loop/loop*^ mutant mice and increased after Betahistine treatment compared with wild-type mice, consistent with the results in mice with gentamicin induced vestibular dysfunction (Fig. 8F–J).

## Discussion

BPPV is caused by undesirable stimulation of vestibular receptors in the semicircular canal [2]. In most cases, its characteristics can easily be interpreted as excitation/inhibition of ampullary vestibular receptors, followed by the inappropriate flow of endolymph associated with abnormal otoconia in semicircular canals [3]. The most common cause of BPPV is tubular stones in posterior canal (PC) and lateral semicircular canals (LC), and treatment is a physical operation that removes (repositions) the auricle from the canal [7, 30].

Several studies shown that Betahistine is effective in reducing the frequency and severity of vertigo and improving vertigo related symptoms. A Cochrane systematic review published in 2016 concluded that the proportion of patients reporting an overall reduction in their vertigo symptoms was higher in the group treated with Betahistine than the placebo group [31]. A study conducted in Russia and Ukraine by Parfenov et al., in which 305 patients were included of which 74% achieved a clinical response considered excellent [31]. In BPPV, repositioning maneuver and Betahistine in BPPV patients reduced the DHI score from 52.16 (range 20–100) before treatment to 17.84 (range 0–78) after treatment, which could effectively improve BPPV [32]. In our study, we found that the duration of dizziness was significantly shortened, the DHI score was significantly reduced and the BBS score was significantly increased in BPPV patients treated with Betahistine.

Reports have shown that gentamicin, as an aminoglycoside, produces ototoxicity and vestibulotoxicity by inducing condensation of the nuclei of outer hair cells followed by loss of mitochondrial membrane potential and apoptosis, while damage to the vestibular apparatus results in dizziness, ataxia, and/or nystagmus [33]. Therefore, we simulated BPPV by establishing a mouse vertigo model of vestibular dysfunction by injecting gentamicin to investigate the effects of Betahistine on BPPV. We found that Betahistine treatment of vertigo mice with vestibular dysfunction increased the accuracy of the air righting reflex, reduced the time of contact the righting reflex, and significantly decreased head tilt and swimming behavior scores, and the same results were observed after Betahistine treatment of the *Slc26a4*^*loop/loop*^ mutant mice. Therefore, our study is consistent with previous reports that Betahistine effectively alleviates BPPV. Those findings documented in previous research and our study can be explained by the mechanism of action of Betahistine, which includes among others a histaminergic vasodilator effect of the cerebral and inner ear microcirculation as well as by its action at the level of the central histaminergic system, which improves the process of vestibular compensation and reduces the spontaneous activity of peripheral vestibular receptors [9].

Several studies have reported that Otolin as a secreted glycoprotein, could regulate BPPV and has been identified as a biomarker for BPPV [34, 35]. CTRP family members have a C-terminal globular domain homologous to Otolin and depend on this domain for biological activity [29, 36, 37]. However, it’s not clear whether CTRPs play a role in BPPV, and therefore, we examined the serum levels of CTRP family members in Betahistine treated BPPV patients. The results showed that serum CTRP 2, 4, 5, 7, 10, 11, 13, 15 and adiponectin levels were increased after betahistine treatment in BPPV patients, whereas CTRP 1, 3, 6, 9 and 12 levels were significantly increased. To verify this phenomenon, we constructed a vertigo mice model of vestibular dysfunction and injected pSB-T-CTRP vector or si-CTRP containing CTRP1, 3, 6, 9 and 12, whose expression levels changed significantly before and after Betahistine treatment of BPPV, respectively. Results showed that overexpression of CTRP1, 3, 6, 9 and 12 could all ameliorate the behavioral abnormalities of mice with vestibular dysfunction vertigo. Silencing CTRP1, 3, 6, 9 and 12 all aggravated behavioral abnormalities in mice with vestibular dysfunction vertigo. Although we avoided their common sequences as much as possible when designing siRNAs for CTRPs, it still failed to completely guarantee their specificity, and there are still individual siRNAs that silence 2 or 3 genes simultaneously. Therefore, in this study, we determined the involvement of CTRPs in vestibular dysfunction, but could not determine whether silencing individual CTRPs equally exacerbates vestibular dysfunction and whether they are connected.

Subsequently, we constructed *Slc26a4*^*loop/loop*^ mutant mice as an animal model of BPPV and observed that in *Slc26a4*^*loop/loop*^ mutant mice, the expression of CTRPs were decreased and increased after Betahistine treatment. Based on the above results, we determined that CTRPs played an important role in the process of Betahistine treatment for BPPV. It has also been reported that in BPPV cases, the otoconia may detached and released, and the recurrence of BPPV is thought to be caused by vascular disease. Betahistine produces vasodilatation and improves microcirculation in the inner ear[9, 38]. These mechanisms may explain the effectiveness of Betahistine as a coordinator of vestibular compensation in recurrent cases of BPPV. However, we did not perform an in-depth study on the effect of Betahistine on vasodilation in this study, so it cannot be determined whether CTRPs play a role in the improvement of microcirculation in the inner ear by betahistine.

Literature showed that CTRP1 of CTRP family members specifically activates AKT and p44/42-MAPK (mitogen-activated protein kinase) signaling pathways in differentiated mouse myotubes to reduce their serum glucose levels [29]. CTRP3 promotes the proliferation of undifferentiated C2C12 myoblasts through activating extracellular signal-regulated kinase 1/2 (ERK1/2) pathway [39]. CTRP6 alleviates AngII-induced hypertension and vascular endothelial dysfunction in spontaneously hypertensive rats through activating PPARγ [14]. CTRP9 significantly increased the expression of adipoR1, PI3K, p-AKT, and Bcl-2, and attenuated neuronal apoptosis after ICH in mice through adipoR1/PI3K/AKT pathway [40]. Overexpression of CTRP12 significantly increased the phosphorylation level of AKT to improve insulin signaling in adipose tissue and liver [41]. In our study, we found that phosphorylation levels of ERK1/2 and AKT were significantly increased and the mRNA and protein expression levels of PPARγ were significantly upregulated after pBS-T-CTRP1 and pBS-T-CTRP12 were injected into vestibular dysfunctional vertigo mice, respectively. It has also been reported that 15 members of the CTRP family have similar structures and play a role in metabolism and immunity [28, 42]. Based on these results, we speculate that CTRP family members have similar effects and that their overexpression might activate the ERK1/2-AKT/PPARγ signaling pathway. In addition, we also observed that Betahistine induced the expression of CTRP1, 12, phosphorylation of ERK1/2 and AKT and the expression of PPARγ, and significantly increased the accuracy of air righting reflex, reduced the time of contact righting reflex and decreased the scores of head tilt and swimming behavior in mice of vestibular dysfunction vertigo. The ERK1/2 inhibitor SCH772984 and PPARγ antagonist GW9662 counteracted the effect of Betahistine in vestibular dysfunction vertigo mice, but had no effect on the expression of CTRP1 and CTRP12. In other words, Betahistine induced the expression of CTRP family members, and overexpression of CTRP family members activates the ERK1/2-AKT/PPARy pathway. However, whether the CTRP family members activate the ERK1/2-AKT/PPARγ pathway by themselves or whether they have reciprocal influences on each other is unknown and awaits further investigation.

## Conclusion

Our studies demonstrated that Betahistine alleviates BPPV through inducing production of multiple CTRP family members and activating the ERK1/2-AKT/PPARy pathway. Our findings provide new ideas and theoretical basis for the treatment of BPPV.

## Supplementary Information

**Additional file 1:** **Table S1.** DHI Questionnaire.

**Additional file 2:** **Figure S1.** The clinical effect of Betahistine on BPPV and the expression of CTRP2, 4, 5, 7, 10, 11, 13, 15 and adiponectin. BPPV patients were treated with Betahistine (12 mg/time, 3 times/day) for 4 weeks, and blood was collected and serum was separated. The expression of CTRP2 (**A**), CTRP4 (**B**), CTRP5 (**C**), CTRP7 (**D**), CTRP10 (**E**), CTRP11 (**F**), CTRP13 (**G**), CTRP15 (**H**), and adiponectin (**I**) in serum was detected by using ELISA. N=5, statistical differences were performed by using the Student’s t-test. Compared with BPPV patient, *P < 0.05, **P <0.01. **Figure S2.** Betahistine reduces vestibular dysfunction through inducing the expression of CTRP family members and activating the ERK1/2-AKT/PPARγ pathway in mice. Fifty-six mice were randomly divided into four groups and treated with normal saline, gentamicin, gentamicin + Betahistine, gentamicin + Betahistine + GW9662, respectively. Then, the mRNA levels of CTRP1 (**A**) and CTRP12 (**B**) were detected with RT-qPCR. We also evaluated the accuracy of air righting reflex (**C**), the time of contact righting reflex (**D**) and the scores of head tilt and swimming behavior (**E**) in mice. N=7, Statistical differences were performed by using a one-way ANOVA. *P < 0.05, **P <0.01.

## Data Availability

The datasets used and/or analyzed during the current study are available from the corresponding author on reasonable request.

## Abbreviations

BPPV: Benign paroxysmal positional vertigo
CTRP: Complement C1q/TNF related protein
pBS-T-CTRP: CTRP expression vector
si-CTRP: Small interfering RNA of CTRP
DHI: The 25-item dizziness handicap inventory
BBS: The modified Berg balance scale
