# Supplementary material for: Betahistine alleviates benign paroxysmal positional vertigo (BPPV) through inducing production of multiple CTRP family members and activating the ERK1/2-AKT/PPARy pathway
Source: Biol Res. 2022 Apr 4;55:16. doi: 10.1186/s40659-022-00385-3 (PMC8981858; doi:10.1186/s40659-022-00385-3)
Supplement: Supplementary file 2 — Additional file 2: Figure S1. The clinical effect of Betahistine on BPPV and the expression of CTRP2, 4, 5, 7, 10, 11, 13, 15 and adiponectin. BPPV patients were treated with Betahistine (12 mg/time, 3 times/day) for 4 weeks, and blood was collected and serum was separated. The expression of CTRP2 (A), CTRP4 (B), CTRP5 (C), CTRP7 (D), CTRP10 (E), CTRP11 (F), CTRP13 (G), CTRP15 (H), and adiponectin (I) in serum was detected by using ELISA. N=5, statistical differences were performed by using the Student’s t-test. Compared with BPPV patient, *P < 0.05, **P <0.01. Figure S2. Betahistine reduces vestibular dysfunction through inducing the expression of CTRP family members and activating the ERK1/2-AKT/PPARγ pathway in mice. Fifty-six mice were randomly divided into four groups and treated with normal saline, gentamicin, gentamicin + Betahistine, gentamicin + Betahistine + GW9662, respectively. Then, the mRNA levels of CTRP1 (A) and CTRP12 (B) were detected with RT-qPCR. We also evaluated the accuracy of air righting reflex (C), the time of contact righting reflex (D) and the scores of head tilt and swimming behavior (E) in mice. N=7, Statistical differences were performed by using a one-way ANOVA. *P < 0.05, **P <0.01. [file 40659_2022_385_MOESM2_ESM.docx]

**Supplemental Figure legends**


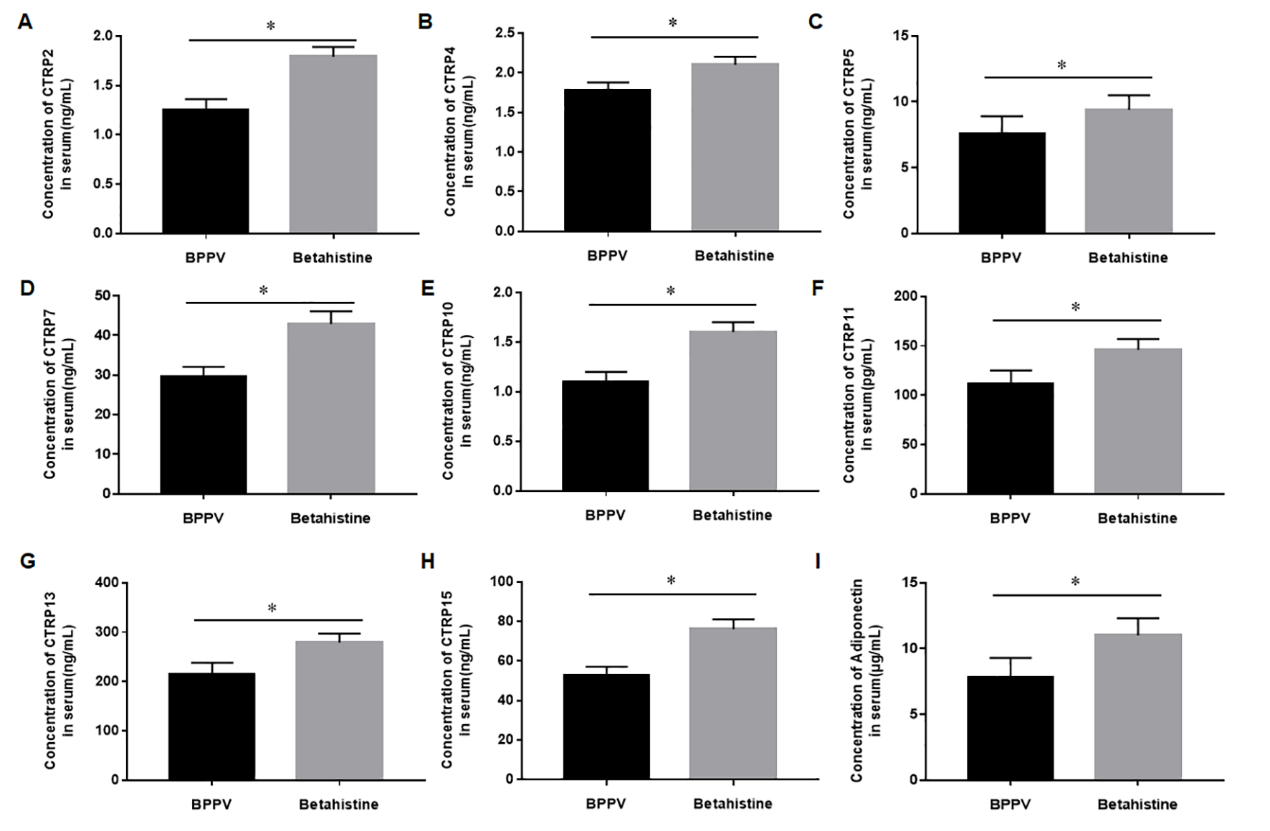


**Supplemental Figure 1:** **The clinical effect of Betahistine on BPPV and the expression of CTRP2, 4, 5, 7, 10, 11, 13, 15 and** **adiponectin.** BPPV patients were treated with Betahistine (12 mg/time, 3 times/day) for 4 weeks, and blood was collected and serum was separated. The expression of CTRP2 (**A**), CTRP4 (**B**), CTRP5 (**C**), CTRP7 (**D**), CTRP10 (**E**), CTRP11 (**F**), CTRP13 (**G**), CTRP15 (**H**), and adiponectin (**I**) in serum was detected by using ELISA. N=5, statistical differences were performed by using the Student’s t-test. Compared with BPPV patient, **P* < 0.05, ***P* <0.01.


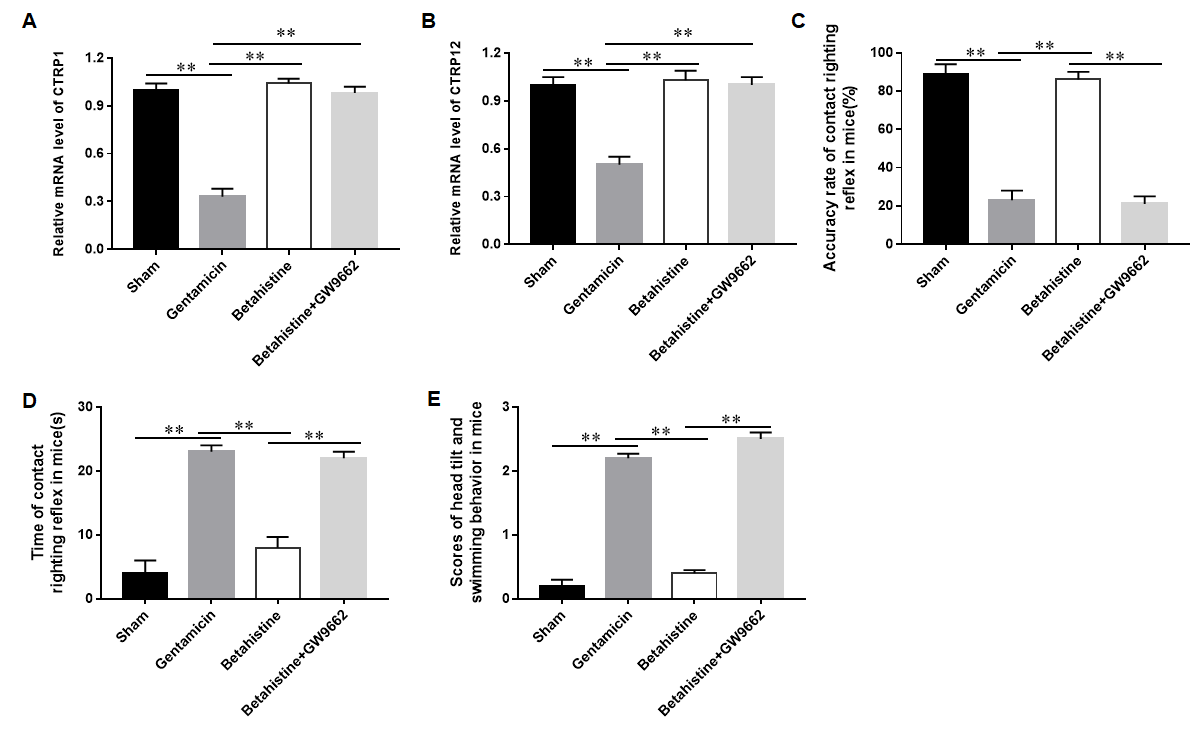


**Supplemental Figure 2: Betahistine reduces vestibular dysfunction through inducing the expression of CTRP family members and activating** **the ERK1/2-AKT/PPARγ pathway in mice.** Fifty-six mice were randomly divided into four groups and treated with normal saline, gentamicin, gentamicin + Betahistine, gentamicin + Betahistine + GW9662, respectively. Then, the mRNA levels of CTRP1 (**A**) and CTRP12 (**B**) were detected with RT-qPCR. We also evaluated the accuracy of air righting reflex (**C**), the time of contact righting reflex (**D**) and the scores of head tilt and swimming behavior in mice (**E**). N=7, Statistical differences were performed by using a one-way ANOVA. **P* < 0.05, ***P* <0.01.

**Supplemental table 1: DHI Questionnaire.**
